# Effect of explicit visual feedback distortion on human gait

**DOI:** 10.1186/1743-0003-11-74

**Published:** 2014-04-28

**Authors:** Seung-Jae Kim, Dieudonne Mugisha

**Affiliations:** 1 Department of Biomedical Engineering, California Baptist University, 8432 Magnolia Avenue, Riverside, CA 92504, USA; 2 Department of Electrical and Computer Engineering, California Baptist University, Riverside, CA 92504, USA; 3 Department of Electrical and Computing Engineering, Utah State University, Logan, UT 84322, USA

## Abstract

**Background:**

Gait rehabilitation often utilizes correction of stepping movements, and visual feedback is one of the interactive forms that can be used for rehabilitation. We presented a paradigm called visual feedback distortion in which we manipulated the visual representation of step length. Our previous work showed that an implicit distortion of visual feedback of step length entails unintentional modulations in the subjects’ gait spatial pattern. Even in the presence of cognitive load through a distraction task, distortion of visual feedback still induced modulation of gait step length. In the current study, subjects were aware of the imposed distortion of visual feedback and they were instructed to maintain their natural gait symmetric pattern during trials. We then studied whether such an explicit “visual feedback distortion” would still influence gait spatial pattern.

**Methods:**

Nine healthy subjects participated in the treadmill walking trial. The step length was defined as the distance between each foot. The on-line visual feedback showing right and left step length information as bar graphs was displayed on a computer screen. When distorting the visual feedback, the height of the bar for only one side was manipulated, so that subjects perceived their step length as being asymmetric. Actual step lengths were measured during trial and analyzed to see the effects of visual feedback distortion.

**Results:**

Our results showed that a gradual distortion of visual feedback systematically modulated gait step length away from symmetry even at the expense of an opposing apparent task goal. It was also observed that the amount of induced gait modulation was reduced during the explicit condition compared to the implicit condition where subjects were not aware of distortion.

**Conclusions:**

Our study demonstrated that although the visual feedback display used in this study did not alter visual space or evoke illusions of motion, perturbation of visual information about subjects’ movement can cause unintentional motor functions. This suggests that the effect of visual feedback distortion is spontaneous and a gait training involving the visual distortion paradigm may provide an effective way to help subjects correct gait patterns by driving implicit motor functions, thereby bringing benefits to rehabilitation.

## Background

Gait restoration is an integral part of rehabilitation for people with brain lesions, spinal cord injuries, and traumatic accidents involving the hip or lower limbs [1-3]. Gait training that uses passive movements or treadmill training with weight-support can provide repetitive stepping movements, thereby promoting activity-dependent plasticity in the central nervous system. This eventually enhances the speed and efficacy of walking in people with disorders [4]. However, the functional gains from gait training have been shown to be moderate. Perhaps this is because the present forms of gait training may not be interactive enough to promote an efficient motor learning process [5,6].

To help enhance the effectiveness of gait rehabilitation, visual feedback can be implemented into the training, so that not only can subjects monitor their own movements, but also keep them engaged. In particular, visual feedback provided during therapeutic training helps subjects become aware of how they move and correct any compensatory strategies, which in turn stimulates the motor learning process and leads to better functional outcomes [7-9]. Furthermore, a paradigm called visual feedback distortion, where visual feedback is distorted or manipulated, has been proposed for rehabilitation therapy with a potential benefit. Brewer et al. [10] showed, in a finger-motion rehabilitation trial, that there was a considerable physical gain in using visual feedback that distorted the information about the force being exerted by a subject’s fingers.

As a prelude to developing such a paradigm for utilizing visual feedback distortion in the context of gait rehabilitation, we have previously proposed a novel and simple method of providing visual feedback of subjects’ step lengths. In the previous study, the right and the left step lengths during treadmill walking were measured and represented by vertical bar graphs. This on-line visual feedback was displayed to subjects. Then, we implicitly distorted the visual feedback of the step lengths while the subjects were unaware of the manipulation. For distortion of visual feedback, we changed the scaling factor between the actual step length and the displaying bar height for only one side of the leg so that subjects perceived their step lengths as being asymmetric. The applied distortion level was adjusted during trial. Our previous study showed that the implicit visual feedback distortion affected gait symmetry. Even with a distraction task added, subjects spontaneously modulated their gait symmetric pattern away from actual symmetry in response to the implicit distortion of visual feedback [11]. This suggests that the effect of visual feedback distortion on changes in step symmetry involves implicit or unintentional resources, but still posed the question whether or not some sort of cognitive action was also employed in the modulation of gait symmetry.

In the current study, we expanded on our study by testing healthy subjects using an explicit condition, where we informed them of the imposed distortion of the visual feedback and also instructed them to maintain their natural step symmetric pattern during treadmill walking. Our hypothesis was that the explicit visual feedback distortion could still affect the stepping pattern of subjects. A positive outcome of this study would suggest the importance of unintentional processes in gait adaptation to visual feedback distortion and the potential use of visual feedback distortion may be of value for gait rehabilitation.

## Methods

### Subject

Nine healthy subjects (five men and four women; 19–41 years of age) participated in the experiment with explicit visual feedback distortion. All of the subjects were familiar with walking on a treadmill, and gave informed written consent before participating. The experimental protocols were approved by the California Baptist University Institutional Review Board.

### General experiment setup

**Figure 1:**
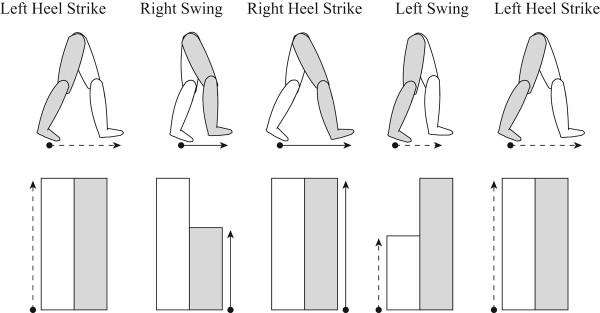
**Visual feedback display.** The range of the right and left step lengths was mapped to the visual feedback bars, shown in the bottom row. For example, during the swing phase of a leg, the corresponding bar increases in real time proportional to the step length and stops when heel-strike occurs for that leg. The range of step length mapped to the visual bar was then gradually distorted.

All of the subjects practiced walking on a treadmill for more than ten minutes and chose their natural walking speed which varied between 2.0 mph and 2.4 mph. They continued to walk on the treadmill with a visual display representing their step lengths. The on-line visual feedback system, placed in front of the treadmill displayed vertical bar graphs corresponding to the subjects’ step length. Step length was defined as the distance between feet and was mapped to the visual feedback bars (Figure 1). During the swing phase of a leg, the step length increases. Likewise, the bar corresponding to the leg in swing phase increases in height. When the heel of the leg strikes the treadmill floor, the bar remains at its maximum for the whole stance phase until the next swing phase begins. Thus, at every heel strike, whether it is from the left or the right leg, it is possible to observe the maximum length of both the right and the left steps side by side. We explained to the subjects the meaning of the bar graphs and they clearly understood it during the initial practice without any distortion.

During the explicit distortion trial, we distorted the length of the right bar in increments of 102%, 104%, 108%, and 116% of the actual step length. For example, 104% distortion changed the scaling factor between the actual right step length and the displayed bar height from 100% to 104%. In other words, a distortion of 104% displays a stride length bar that is 1.04 times the height of the actual stride. In this way, subjects visually perceived that their right step length was 4% longer than the actual length during visual distortion. All of the subjects were clearly informed that the visual representation of their right step length would be distorted in such a way and we instructed them to maintain their natural step symmetric pattern during the trial. Subjects were able to distinguish the distortion from their own variability at 104% and greater distortion levels.

Actual step length was measured using an optometric tracking system (OPTOTRAK system, Northern Digital Inc., Canada). Two infrared markers were attached on the heel sides of the subjects’ shoes. The markers emit infrared light that is seen by a 3-D camera to locate their position. The position data of the markers was retrieved by a PC in real time using a program constructed within LabVIEW to graphically represent the step length measurements.

### Procedure

**Figure 2:**
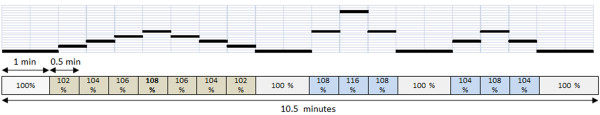
**Distortion profiles applied during the trial.** The experimental trial lasted 10.5 minutes, starting and ending with one minute of walking without distortion. Each distortion level lasted for 30 seconds. The rate changes in distortion level were 2%, 8%, and 4% in the first, the second, and the third block periods, respectively. The horizontal bars graphically represent the change of distortions over time.

The experimental trial lasted 10.5 minutes. While walking on the treadmill, subjects continued to look at the visual feedback display over the entire period. During each trial, three different profiles of distortion adjustment were applied to see the effects of changing the rate of distortion level on the change in step length symmetry (Figure 2). The periods over which different distortion profiles were applied were referred to as block 1, block 2, and block 3, respectively. The entire 10.5 minute trial started and ended with one minute of no-distortion period (100% distortion level); each block in the trial was also separated by one minute of no-distortion period. In the first block, the distortion was increased by increments of 2% up to 108%, and then decreased by the same rate down to 100%. In the second block, the distortion was increased by increments of 8% up to 116% and then reduced by the same scale down to 100%. In the last block, the distortion increased by 4% up to 108% and then decreased down to 100% with the same rate. Each distortion level lasted for 30 seconds. Subjects practiced walking on a treadmill for more than ten minutes to familiarize themselves with walking on a treadmill while looking at a computer screen.

### Data analysis

The measurement of interest in this study was the step length symmetry. This value was calculated as the ratio (%) between the left step length and the right step length calculated as Step Length Symmetry (%) =100 * (the left step length/the right step length). Thus, a step symmetry value smaller than 100% meant that the right step was longer than the left step and vice versa for values greater than 100%. The mean step symmetry values (the symmetry metric) over distortion levels (with 30-second period at each level) were calculated per subject and used to test the changes in step symmetry induced by the explicit visual distortion. The repeated-measured Analysis of Variance (ANOVA) was employed to examine whether there was a change in the symmetry metric for different visual distortion levels. With paired t-test, we also examined the minimum distortion level that had induced significant changes in step symmetry from the initial 100% period.

In the current study, we tried to compare the extent of gait modulation in response to the explicit and implicit distortion for further discussion. For the purpose of this comparison, we used the naïve (implicit) group data from our previous study [11]. The naïve group refers to the group tested under implicit visual distortion condition where subjects were unaware of the imposed distortion of visual information about their step length during the trial. Because the experimental procedures were not identical between the two conditions (explicit or informed group vs. implicit or naïve group), the comparison was done only for the period when the distortion level was increased from 100% to 108%. We employed two separate analyses using a two-factor ANOVA comparing the two data sets to examine whether there was a significant effect of distortion level and instruction (explicit or implicit) on the changes in step length symmetry. The first comparison was performed with the ‘naïve with distraction’ with ‘explicit (informed)’. The second comparison used the ‘naïve with no-distraction’ with ‘explicit (informed)’.

## Results

**Figure 3:**
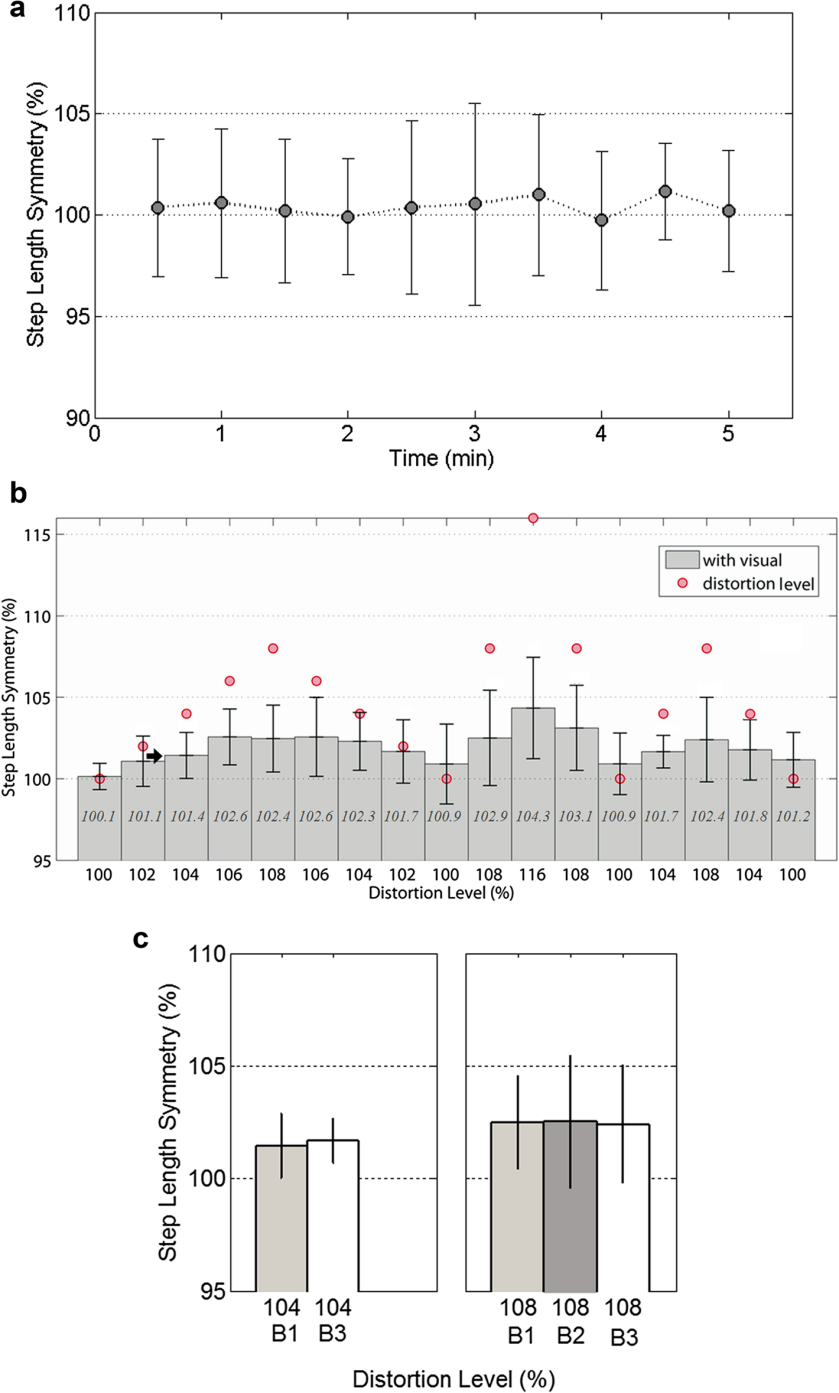
**Average step length symmetry over time. (a)** Example of changes in step length symmetry during 5-minute treadmill walking with no visual feedback. **(b)** Changes in the mean step length symmetry as a function of varying visual feedback averaged across all of the subjects during the explicit visual distortion condition (informed group). **(c)** Effect of the changing rate of distortion level on change in step length symmetry (adapted from Figure 3b).

Figure 3a shows an example of the variability in step length symmetry over a five minute period of normal walking on a treadmill. The data was obtained from a control setting where subjects walked on the treadmill without visual feedback. The horizontal axis corresponds to time and the vertical axis represents step symmetry ratio. The solid circles represent the mean step symmetry value, averaged across nine subjects over subsequent 30 seconds intervals. Error bars indicate ± one standard deviation (σ). The changes of step length symmetry indicate a small degree of fluctuation over time. For instance, the lowest value was 99.9% while the highest value was 101.2%. The highest standard deviation was 4.7%. No pattern was found in the changes of step length symmetry over the five minutes of normal walking.

In order to investigate how the explicit visual feedback distortion affected the stepping pattern of subjects, we plotted the acquired data during the 10.5-minute session of visual feedback distortion. The entire session contains three block periods (B1: 2% change of distortion level, B2: 8% change, and B3: 4% change) depending on different increments/decrements of distortion. Figure 3b shows the changes in step length symmetry under the explicit visual distortion, averaged across nine subjects, as a function of varying feedback distortion. The horizontal axis shows changes in the distortion level and the vertical axis shows the step length symmetry. The solid bars show the percentage of mean symmetry where 100% is the perfect symmetry between the left and the right step lengths. The thin vertical lines show the standard deviation (σ) among the nine subjects for each distortion level. The solid circle show the distortion level applied at given periods. The solid arrow was marked at the distortion level where the induced step symmetry values were first found to be significantly different from the initial 100% period (p < 0.001). From the data in Figure 3b, it was observed that the mean step length symmetry increased and then decreased according to the imposed distortion levels. For instance, the highest distortion (116%) affected the step symmetry the most yielding an induced gait asymmetry value of 104.3%. The repeated-measures ANOVA test showed significant effects of visual distortion on the dependent variable (step length symmetry), *F(4.196, 33.371) = 2.818, p < 0.05*. Significant changes in step length symmetry were found at as low as 104% distortion level (marked by the solid arrow in figure) compared to the initial 100% baseline. P-values from pairwise t-test between the initial step length symmetry and the step length symmetry at each different distortion level (102%, 104%, 106%, 108%, 106%, 104%, 102%, 100%, 108%, 116%, 108%, 100%, 104%, 108%, 104%, and 100%) over the entire trial were 0.0135, <0.0001, 0.0003, 0.0215, 0.0287, 0.0132, 0.0340, 0.4619, 0.0481, 0.0019, 0.0218, 0.3169, 0.0096, 0.0613, 0.1135, and 0.1872, respectively. These results indicate that subjects were unable to sustain explicit control of maintaining natural symmetric gait pattern in response to visual feedback distortion.

Figure 3c shows the effect of the increased rate of distortion level on the change in step length symmetry. The bars represent the mean symmetry values at the same distortion levels (104% and 108%) in three different blocks (B1: 2% change of distortion level, B2: 8% change, and B3: 4% change) over the distortion-increasing session. Thus, this plot simply shows the mean step length symmetry values in different blocks (B1, B2, and B3) at the same distortion levels during the period when the distortion increased only. It appeared that the rate of change of distortion level did not affect the change in gait modulation. The ANOVA test showed no significant differences.

## Discussion

### Effects of distorting visual feedback on step length symmetry

The visual system is well integrated with the control of human locomotion by providing information about the walking environment and also proprioception through the environment [12]. Many studies using virtual reality technology have shown that changing the speed of virtual environment affects balance and walking speed [13-15], suggesting an intrinsic link between locomotion and visual perception. In the current study, we demonstrated that gradual distortion of visual feedback of step symmetry, even with explicit knowledge of the manipulation, systematically modulated gait step length away from symmetry. These results reinforce the fact that changes in visual perception influence correction of walking movement patterns. We created visual information using very simple bar graphs that represent the right and the left step length in the context of treadmill walking (Figure 1). Then, we distorted the visual information provided to healthy subjects in a way so that they perceived their step symmetric pattern as being asymmetric. The method used in our study is distinct from other visuomotor adaptation studies, where the whole visual scene of artificial environments was controlled by virtual reality technology. Despite the fact that the visual representation used in our study did not evoke any illusion in the visual field on the retina, all of the subjects spontaneously modulated their gait step pattern according to the visual feedback distortion.

In our previous study, we implicitly distorted the visual information. We told subjects to walk normally and they were not aware of the imposed distortion of visual information about their step length. Subjects spontaneously modulated their spatial gait pattern to compensate for the asymmetric representations of their step lengths. They were also completely unaware of the nature of their gait modulation induced by the visual distortion. However, since such effects of visual feedback distortion might entail subjects’ conscious-compensating processes for the asymmetric representations of step length, we included a distraction task that might help to remove subjects’ volitional or cognitive control to some extent. Although the amount of the induced step symmetry was reduced during the distraction task, we found that visual feedback distortion still influenced step symmetry. These findings imply that the visual feedback distortion used in our study entails unintentional or implicit processes. However, we could not eliminate the possibility that the effect was still partly the consequence of any remaining cognitive or conscious strategy because the distraction task might not have removed the subjects’ potential conscious control completely.

### Effects of explicit visual feedback distortion on gait symmetry

The current experiment sought to investigate the effect of explicit visual feedback distortion on gait modulation. The experimental paradigm was to place explicit strategy during treadmill walking under visual feedback distortion. We informed subjects of the imposed distortion of visual feedback of step lengths and told them not to be misled by the visual feedback and also to maintain their natural gait symmetric pattern. We investigated the role of explicit visual information in spatial gait pattern using a paradigm of visual feedback distortion. Surprisingly, despite subjects’ explicit strategy, the visual feedback distortion influenced them to spontaneously modulate the gait step symmetry (Figure 3b). When the distortion level was varied systematically, the amount of the induced gait modulation changed accordingly. Subjects were interviewed after the experiment, and they described that they were unaware of the changes in their gait symmetry during the trial.

The lower limbs and their movement during walking are essentially bilaterally symmetrical perhaps due to inherently symmetrical organization of neural circuits within the nervous system [16]. Proprioceptive feedback from the lower limbs plays an important role in gait symmetry control [17,18]. However, the observation that subjects were unable to maintain the same motor command under the condition of explicit visual distortion implies that the role of supraspinal function through visual system may be a dominant form of feedback in gait control [19]. It should also be noted that the characteristics of supraspinal, e.g., visual influence, are not necessarily confined to conscious or voluntary action.

Visual influence on human locomotion has been previously reported in the field of gait and posture [12-15,19,20]. In these studies, a wedge prism model or artificial optic flow patterns were used to alter the sensation of self-motion of subjects through the environments. For example, since light passing through the prism is bent, a wedge prism causes the visual world to appear displaced to one side of where it would normally appear without the prism. For another technique, artificial optic flow patterns are projected onto a large screen, evoking the illusion of motion along the line of walking. During locomotion, adaptation induced by such visual perturbations in general may be attributed to an interaction between two different modalities, visual and proprioceptive information [21]. The visual perception of motion and/or position may play a proprioceptive role, thereby interacting with other proprioceptive information and modifying locomotion activity on a spinal level [14]. However, there are crucial differences between these studies and the current one. The visual feedback used in our study did not generate the visual perception of motion or position at all. It simply provided subjects with the visual information about their step lengths. Nevertheless, subjects were not able to perform the task, which was to maintain natural symmetric gait pattern, when the visual feedback distortion was imposed.

Although it remains unclear about the specific characteristics of supraspinal involvements through visual system in the context of our experiments, our speculation is not all knowledge about the self-walking motion comes through our physical senses. Some of it comes through our innate knowledge about the ego-motion, and the notion of walking symmetry may be a component of our physical being because we have been walking in such a fashion most of our lives. If perception through visual feedback appears to differ from that knowledge, gait adaptation would occur in order to compensate for the discrepancy even if the visual information is not something like what can control optic flow or evoke illusions. Another explanation of this may be that the motor system cannot tolerate the conflict, in visual space, between the planned trajectory through a prediction of motor circuits and executed trajectory perceived in a visual space by visual feedback [22]. Thus, such conflict may drive implicit adaptation, in opposition to the explicit strategy.

### Effects of increasing rate of distortion level on the amount of induced gait symmetry

In our experiment, the visual distortion level went up by three different increments; 102%, 104%, and 108% (Figure 2). It appeared that a small change of the distortion level, such as 102% change of step length mapped to the visual bar, was hardly noticed by subjects, whereas larger distortion levels higher than 2% were easily noticed. Thus, we examined the effects of the increasing rate of distortion level on the amount of induced step symmetry (Figure 3c). During the period when the distortion was increased, there were no significant differences in the amount of induced gait symmetry among different rate changes of distortion level. Although the evidence is not yet conclusive, this observation indicates that the extent of gait modulation induced by visual distortion may be independent of the rate changes of distortion level. This may also imply that the induced gait modulation bears on unintentional or implicit strategy because the amounts of induced gait asymmetry were similar at the same distortion levels regardless of whether or not subjects were able to easily notice the change of the distortion.

### Comparison of visual distortion effects between implicit and explicit conditions

**Figure 4:**
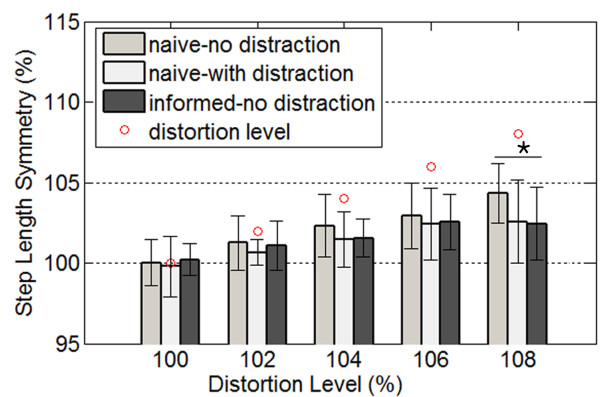
**Changes in step lengthy symmetry under three different conditions (naïve with no distraction, naïve with distraction, and informed with no distraction) during the period when the distortion was increased (Naïve group data was cited from our previous study **[11]**).** Subjects (n = 12) in naïve group were not aware of the distortion of visual feedback whereas the informed group (n = 9) were aware of it during the trial. The distraction task helped remove subjects’ cognitive action. The vertical lines show the standard deviation among subjects for each distortion level. The small circles indicate distortion changes applied during the trial. The asterisks (*) were marked at distortion levels where the induced step symmetry values were shown to be significantly different between different conditions *(p = 0.041).*

In order to understand how the explicit visual distortion influenced the gait symmetry differently from the implicit visual distortion, we tried to compare our results with those obtained from our previous study in which subjects were not aware of the distortion [11]. The implicit condition was again divided into two different conditions (no-distraction and with-distraction conditions). The distraction task consisted in asking subjects to subtract 7 out loud starting at 1000 [23]. Figure 4 shows the changes in step length symmetry under three different conditions (naïve with no distraction, naïve with distraction, and informed with no distraction) during the period when the distortion was increased only. Since the applied distortion profile used for the explicit condition (informed with no distraction) was not identical to that used for the implicit one (naïve group) from the previous study, only the results from the same distortion levels were compared among three different conditions. The naïve group refers to the group tested under the implicit visual distortion condition. Subjects were not informed of the manipulation of visual feedback under the implicit condition whereas the informed group were aware of the imposed visual distortion during each trial. The data for the naïve group (n = 12) was cited for the purpose of the comparison from our previous study [11].

As shown in Figure 4, although subjects did not intentionally correct their stepping movements according to the distorted visual feedback, the induced step asymmetry under the implicit condition (naïve with no distraction) was observed to be slightly greater than under the explicit condition. This discrepancy might have been significant at higher distortion level considering that the t-test found a significant difference at the level of 108%. This suggests that the effect of the implicit visual feedback distortion without distraction may involve some cognitive functions, which is not necessarily confined to conscious or voluntary action. It should be also noted that the amounts of induced gait asymmetry induced by the explicit condition and the implicit condition combined with distraction (naïve with distraction) appeared to be similar. There were no significant differences found in step length symmetry between these two conditions. This observation corroborates that unintentional or implicit adaptation processes were driven by the explicit visual feedback distortion.

### Utilizing visual feedback distortion as a rehabilitation intervention

Understanding the adaptation for locomotion is important for rehabilitation. In therapeutic training settings, patients are commonly instructed to consciously correct or change their walking pattern after an injury. Motor learning in such an explicit manner may result in more transient changes than those driven in an implicit manner [24]. With the realization that the visual feedback distortion we provided here can drive implicit gait adaptation, the potential of using such systems for the future gait therapy can be highlighted because it may not only produce longer retention of motor learning after training but also help subjects’ execute movements beyond their volitional efforts.

## Conclusions

The present study investigated the effect of an explicit visual feedback distortion paradigm on influencing modulation of step length symmetry (a spatial gait pattern). The visual information used in this study was simple bar graphs, not altering visual space nor evoking illusions. Subjects were told to maintain their natural gait symmetric pattern and also informed of the distortion of visual feedback. The results of this study showed that subjects spontaneously modulated their gaits’ symmetric pattern away from symmetry according to the distortion of visual feedback even at the expense of an opposing explicit task goal. The effect of visual feedback distortion on changes in step symmetry appears to involve unintentional or implicit motor functions that may be enhanced by conscious involvement. These results suggest that the effect of visual feedback during gait is unnoticed and spontaneous and also that a paradigm of using such visual feedback distortion may be potentially useful as a supplemental therapeutic intervention for gait rehabilitation.

## Competing interests

The authors declare that they have no competing interests.

## Authors’ contribution

SK carried out the experiments and drafted the manuscript. DM helped to collect and analyze experimental data. Both authors read and approved the final manuscript.
